# Thermal Ablation Versus Wedge Resection for Stage I Non-small Cell Lung Cancer Based on the Eighth Edition of the TNM Classification: A Population Study of the US SEER Database

**DOI:** 10.3389/fonc.2020.571684

**Published:** 2020-10-14

**Authors:** Chenxi Zeng, Jiawei Lu, Yitao Tian, Xiangning Fu

**Affiliations:** Thoracic Surgery Laboratory, Department of Thoracic Surgery, Tongji Hospital, Tongji Medical College, Huazhong University of Science and Technology, Wuhan, China

**Keywords:** thermal ablation, wedge resection, NSCLC, stage I, survival, SEER

## Abstract

**Background:**

The incidence rates of early-stage non-small cell lung cancer (NSCLC) are now increasing, and therapies such as thermal ablation have shown potential therapeutic promise. This study aimed to determine the influence of different surgical methods on overall survival (OS) and cancer-specific survival (CSS) in patients with stage I NSCLC.

**Methods:**

Patients diagnosed with stage I NSCLC who had received thermal ablation or wedge resection between 2004 and 2014 were obtained from the Surveillance, Epidemiology, and End Results (SEER) database. Propensity score matching (PSM) was performed according to the surgical method. Kaplan–Meier curves and a Cox proportional hazard model were used to evaluate OS and CSS.

**Results:**

In all, 4,372 patients with stage I NSCLC were included. Before PSM, the respective 3- and 5-year OS rates were 68.9 and 52.7% in the wedge resection group and 68.5 and 47.8% in the thermal ablation group (*p* < 0.0001); the corresponding CSS rates were 79.1 and 69.4% and 62.6 and 46.0% (*p* < 0.0001). After PSM, survival analysis showed that wedge resection had better OS (44.5% vs. 30.1%, *p* = 0.033) and CSS (63.5% vs. 46%, *p* = 0.038) than thermal ablation. After PSM, Cox regression showed that treatment was not associated with OS or CSS. For patients aged >75 years, thermal ablation showed similar OS and CSS as wedge resection (OS: 30.6% vs. 41.7%, *p* = 0.470; CSS: 46.4% vs. 64.1%, *p* = 0.100). After PSM, thermal ablation still had OS (30.6% vs. 41.0%, *p* = 0.470) and CSS (46.4% vs. 59.8%, *p* = 0.100) comparable to wedge resection.

**Conclusion:**

For patients with stage I NSCLC who are unfit for lobectomy, thermal ablation could be a potential therapeutic option, especially for those >75 years old.

## Introduction

Surgical resection is the current standard treatment for patients with early-stage non-small cell lung cancer (NSCLC) (1–5). A randomized controlled trial in 1995 showed that lobectomy could be recommended as the standard surgical procedure for stage I (≤3 cm) NSCLC. Patients who cannot tolerate radical surgery are frequently older patients with comorbidities or impaired pulmonary function. Several studies have reported that segmentectomy and wedge resection show similar survival rates for patients who are unfit for lobectomy (6–9). Additionally, although wedge resection is a non-anatomical resection, it may be optimal for patients who cannot undergo lobectomy.

Image-guided percutaneous radiofrequency ablation using lasers and electrocautery have been developed as ablative therapeutic techniques for local tissue destruction (10). Thermal ablation is regarded as a promising treatment for small tumors in patients unfit for surgery (11, 12). However, it remains unclear whether patients unfit for lobectomy can benefit from wedge resection or thermal ablation.

In this study, we used the large Surveillance, Epidemiology, and End Results (SEER) database to compare the outcomes of patients with stage I NSCLC after wedge resection or thermal ablation, based on the 8th edition of the TNM classification.

## Materials and Methods

### Data Source

We obtained access to the SEER database in November 2019. Our study population included 4,372 patients diagnosed with stages IA and IB NSCLC who underwent thermal ablation or wedge resection between 2004 and 2014. Patients meeting the following criteria were considered eligible for inclusion: (i) histology was identified by ICD-O-3 codes 8140, 8070, 8046, 8250, 8560, 8071, 8012, 8480, 8072, 8481, 8490, 8570, 8255, 8550, or 8260 (13); (ii) no metastasis to the lymph node or other organs; (iii) only one primary tumor; (iv) pathologically confirmed stage IA or IB based on the 8th edition of the TNM classification; and (v) those who underwent thermal ablation or wedge resection. Thermal ablation included laser ablation, cautery, and fulguration in the SEER database. Patients with survival months <1 were excluded from the study. Patients were divided into the thermal ablation and wedge resection groups. Demographic data included age at diagnosis; sex; ethnicity (white, black, Asian, Pacific Islander, or others); and marital status. The cancer characteristics included tumor side (left or right); site (upper lobe, middle lobe, lower lobe, unknown); tumor size (≤1 cm, >1 cm but ≤2 cm, and >2 cm but ≤4 cm); histology (squamous cell carcinoma, adenocarcinoma, or others); and grade (I/II, III/IV, or unknown). Treatment characteristics included lymph node resection and adjuvant therapy (radiotherapy or chemotherapy and none). Survival characteristics included survival months, vital status, and cancer-specific death. Overall survival (OS) and cancer-specific survival (CSS) were derived from the above variables.

### Statistical Analysis

Patients were divided into two groups. Categorical variables were presented as frequencies with percentage *n* (%). Chi-square test or Fisher’s exact test was used to compare these variables between the two groups. Propensity score matching (PSM) was performed using 1:1 nearest neighbor matching with a caliper of 0.001 to obtain a matched pair between the thermal ablation and wedge resection groups. Age at diagnosis (<75 years or ≥75 years), sex, marital status, ethnicity, tumor side and site, tumor size, and histology were used in PSM. Kaplan–Meier (KM) curves for OS and CSS were generated for patients and across strata. Differences between strata were determined by log-rank tests. Cox proportional hazard models were used to assess the relationship of interested variables with OS or CSS. Variables with *p* < 0.1 in the univariate models were included in the multivariate model. *P* < 0.05 was considered to indicate statistical significance. Chi-square test, Fisher’s exact test, and Cox analyses were performed with SPSS Statistics 25.0. KM curves were plotted in R (v3.6.2).

## Results

### Patients Characteristics

In all, 4,372 patients who received thermal ablation or wedge resection for stage I NSCLC were identified from the SEER database. Patients were divided into two groups based on whether they underwent thermal ablation (*n* = 108) or wedge resection (*n* = 4264). In the thermal ablation group, 90 patients underwent laser ablation and 18 underwent cautery or fulguration (data not shown). The clinical characteristics of patients are listed in Table 1. Patients in the thermal ablation group were diagnosed when older (>75 age) (47.2%); were less likely to be married (58.3%); had a histology of squamous cell carcinoma (38.0%); had a lower percentage of grade I/II (30.9%) tumors and lymph node resection (6.5%); and had a higher incidence of adjuvant therapy (23.1%) than those in the wedge resection group. After 1:1 matching of patients based on their respective propensity to undergo thermal ablation, a cohort of 206 patients was selected. There were no significant intergroup differences with respect to any clinical variable.

**TABLE 1:** Characteristics of stage I NSCLC patients from SEER Database from 2004–2014.

Variables	Before PSM		*P*-value	After PSM		*P*-value
*n* (%)	Thermal ablation	Wedge resection		Thermal ablation	Wedge resection	
	*n* = 108	*n* = 4264		*n* = 108	*n* = 108	
**Age at diagnosis**			**0.001**			0.414
≤75	57 (52.8)	2880 (67.5)		57 (52.8)	50 (46.3)	
>75	51 (47.2)	1384 (32.5)		51 (47.2)	58 (53.7)	
**Sex**			0.87			0.680
Male	58 (44.4)	1929 (45.2)		58 (44.4)	40 (40.7)	
Female	60 (55.6)	2335 (54.8)		60 (55.6)	64 (59.3)	
**Race**			0.524			0.762
White	94 (87.0)	3699 (86.7)		94 (87.0)	96 (88.9)	
Black	8 (7.4)	368 (8.6)		8 (7.4)	8 (7.4)	
Asian or Pacific islander	4 (3.7)	168 (3.9)		4 (3.7)	4 (3.7)	
Others	2 (1.9)	29 (0.7)		2 (1.9)	0 (0.0)	
**Marital status**			**0.019**			0.677
Yes	45 (41.7)	2265 (52.9)		45 (41.7)	41 (38.0)	
No	63 (58.3)	1999 (47.1)		63 (58.3)	67 (62.0)	
**Side**			0.890			1.000
Right	63 (58.3)	2459 (57.6)		63 (58.3)	64 (59.3)	
Left	45 (41.7)	1805 (42.4)		45 (41.7)	44 (40.7)	
**Site**			0.435			0.804
Upper lobe	62 (57.4)	2768 (65.0)		62 (57.4)	57 (52.8)	
Lower lobe	38 (35.2)	1260 (29.4)		38 (35.2)	41 (38.0)	
Middle lobe	6 (5.6)	182 (4.3)		6 (5.6)	6 (5.6)	
Unknown	2 (1.9)	54 (1.3)		2 (1.9)	4 (3.7)	
**Tumor size**			0.115			0.853
≤1 cm	9 (8.3)	640 (15)		9 (8.3)	8 (7.4)	
>1 cm and ≤2 cm	55 (50.9)	2150 (50.4)		55 (50.9)	52 (48.1)	
>2 cm	44 (40.7)	1474 (34.6)		44 (40.7)	48 (44.4)	
**Histology**			**0.011**			0.120
Squamous cell carcinoma	41 (38.0)	1187 (28.0)		41 (38.0)	32 (29.6)	
Adenocarcinoma	47 (43.5)	2472 (58.0)		47 (43.5)	62 (57.4)	
Others	20 (18.5)	605 (14.2)		20 (18.5)	14 (13.0)	
**Grade**			**0.000**			0.402
Grade I/II	33 (30.6)	2160 (61.2)		33 (30.6)	32 (29.6)	
Grade III/IV	20 (18.5)	1355 (31.8)		20 (18.5)	28 (25.9)	
Unknown	55 (50.9)	299 (7.0)		55 (50.9)	48 (44.4)	
**Lymph node resection**			**0.000**			1.000
Yes	7 (6.5)	2368 (55.5)		7 (6.5)	8 (5.6)	
No	101 (93.5)	1896 (44.5)		101 (93.5)	102 (94.4)	
**Adjuvant therapy**			**0.000**			0.081
Radiation	14 (13.0)	240 (5.6)		14 (13.0)	6 (5.6)	
Chemotherapy	4 (3.7)	173 (4.1)		4 (3.7)	10 (9.3)	
Both	7 (6.5)	73 (1.7)		7 (6.5)	4 (3.7)	
No	83 (76.9)	3778 (88.6)		83 (76.9)	88 (81.5)	

*Bold *P*-value denotes *p* < 0.05.*

### Survival Analysis in OS and CSS

**FIGURE 1:**
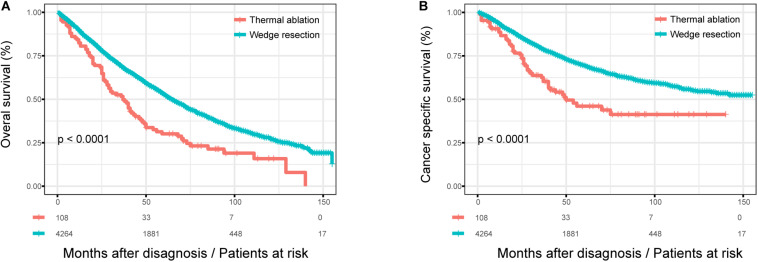
The OS and CSS curves in stage I NSCLC patients who received thermal ablation or wedge resection. **(A)** The OS curve of stage I NSCLC patients who received thermal ablation or wedge resection (χ^2^ = 26.247, *p* < 0.0001). **(B)** The CSS curve of stage I NSCLC patients who received thermal ablation or wedge resection (χ^2^ = 22.453, *p* < 0.0001).

The OS of patients with wedge resection was better than those with thermal ablation (log rank test, *p* < 0.0001) (Figure 1A). The estimated 3- and 5-year OS rates were 68.9 and 52.7% in the wedge resection group, respectively, with a median OS of 65 months (95%CI: 62–67). The corresponding rates were 68.5 and 47.8% in the thermal ablation group with a median OS of 37 months (95%CI: 28–46). Meanwhile, the CSS of patients with wedge resection was also better than those with thermal ablation (log rank test, *p* < 0.0001) (Figure 1B). The estimated 3- and 5-year CSS rates were 79.1 and 69.4%, respectively, in the wedge resection group with no reach for median CSS, while the corresponding rates were 62.6 and 46.0% in the thermal ablation group with a median CSS of 50 months (95%CI: 23–76). The results of the Cox analysis of OS and CSS are shown in Table 2. In the multivariate Cox analysis, treatment was an independent prognostic factor associated with OS and CSS. The thermal ablation group had worse OS (HR: 1.266, 95%CI: 1.003–1.597) and CSS (HR: 1.403, 95%CI: 1.039–1.859) than the wedge resection group. We found that patients who received radiation had worse OS (HR: 1.309, 95%CI: 1.119–1.531) and CSS (HR: 1.309, 95%CI: 1.119–1.531) than those who did not receive adjuvant therapy. Meanwhile, patients who received both radiation and chemotherapy also had worse OS (HR: 1.323, 95%CI: 1.023, 1.711) and CSS (HR 1.730, 95%CI: 1.271, 2.354) than those who did not receive any adjuvant therapies. Other co-variables such as age at diagnosis, sex, tumor size, histology, grade, and lymph node resection were associated with OS and CSS.

**TABLE 2:** Univariate and multivariate Cox regression of OS and CSS in stage I NSCLC patients having thermal ablation and wedge resection.

	Overall survival				Cancer specific survival			
	Univariate		Multivariate		Univariate		Multivariate	
Variables	HR (95%CI)	*P*-value	HR (95%CI)	*P*-value	HR (95%CI)	*P*-value	HR (95%CI)	*P*-value
**Age**								
≤75	Reference				Reference			
>75	1.661 (1.530−1.802)	0.000	1.491 (1.371−1.622)	0.000	1.398 (1.250−1.565)	0.000	1.290 (1.149−1.448)	0.000
**Sex**								
Female	Reference				Reference			
Male	1.427 (1.317−1.546)	0.000	1.301 (1.199−1.411)	0.000	1.381 (1.239−1.540)	0.000	1.260 (1.128−1.407)	0.000
**Race**								
Others	Reference				Reference			
White	1.600 (0.860−2.979)	0.138			1.782 (0.740−4.289)	0.198		
Black	1.410 (0.746−2.664)	0.290			1.798 (0.734−4.401)	0.199		
Asian or Pacific islander	0.914 (0.496−1.873)	0.914			1.144 (0.450−2.908)	0.777		
**Marital status**								
No	Reference				Reference			
Yes	0.946 (0.873−1.025)	0.172			0.985 (0.883−1.098)	0.782		
**Side**								
Left	Reference				Reference			
Right	0.990 (0.913−1.074)	0.806			1.015 (0.909−1.134)	0.785		
**Site**								
Unknown	Reference				Reference			
Upper lobe	0.965 (0.693−1.343)	0.833			0.995 (0.624−1.588)	0.984		
Lower lobe	0.988 (0.707−1.381)	0.944			1.804 (0.676−1.738)	0.738		
Middle lobe	0.981 (0.628−1.342)	0.659			0.951 (0.558−1.621)	0.853		
**Tumor size**								
>2 cm	Reference				Reference			
≤1 cm	0.454 (0.396−0.522)	0.000	0.527 (0.457−0.608)	0.000	0.440 (0.364−0.531)	0.000	0.519 (0.427−0.631)	0.000
>1 cm and ≤2 cm	0.661 (0.607−0.719)	0.000	0.708 (0.649−0.773)	0.000	0.631 (0.563−0.708)	0.000	0.699 (0.620−0.787)	0.000
**Histology**								
Others	Reference				Reference			
Squamous cell carcinoma	1.237 (1.902−1.402)	0.001	1.146 (1.010−1.299)	0.034	1.108 (0.861−1.203)	0.837	0.959 (0.810−1.135)	0.624
Adenocarcinoma	0.781 (0.694−0.879)	0.000	0.869 (0.770−0.980)	0.023	0.750 (0.642−0.875)	0.000	0.851 (0.727−0.997)	0.045
**Grade**								
Unknown	Reference				Reference			
Grade I/II	0.790 (0.683−0.914)	0.002	0.880 (0.755−1.027)	0.104	0.730 (0.593−0.875)	0.001	0.854 (0.694−1.049)	0.133
Grade III/IV	1.183 (1.108−1.375)	0.028	1.178 (1.006−1.378)	0.042	1.156 (0.947−1.410)	0.153	1.225 (0.993−1.512)	0.058
**Lymph node resection**								
No	Reference				Reference			
Yes	0.656 (0.605−0.711)	0.000	0.684 (0.630−0.743)	0.000	0.653 (0.585−0.728)	0.000	0.677 (0.605−0.757)	0.000
**Adjuvant therapy**								
No	Reference				Reference			
Radiation	1.459 (1.248−1.705)	0.000	1.309 (1.119−1.531)	0.001	1.880 (1.551−2.280)	0.000	1.687 (1.390−2.048)	0.000
Chemotherapy	1.173 (0.972−1.414)	0.096	1.041 (0.860−1.260)	0.681	1.827 (1.471−2.269)	0.000	1.551 (1.242−1.938)	0.000
Both	1.706 (1.325−2.196)	0.000	1.323 (1.023−1.711)	0.033	2.344 (1.734−3.170)	0.000	1.730 (1.271−2.354)	0.000
**Treatment**								
Wedge resection	Reference				Reference			
Thermal ablation	1.754 (1.409−2.184)	0.000	1.266 (1.003−1.597)	0.047	1.940 (1.466−2.568)	0.000	1.403 (1.039−1.859)	0.027

*OS, overall survival; CSS, cancer specific survival; HR, hazard ratio; CI, confidence interval.*

### Survival Analysis in OS and CSS After PSM

**FIGURE 2:**
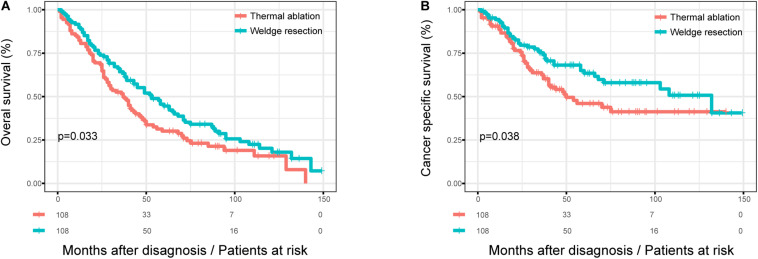
The OS and CSS curves in stage I NSCLC patients who received thermal ablation or wedge resection after PSM. **(A)** The OS curve of stage I NSCLC patients who received thermal ablation or wedge resection after PSM (χ2 = 4.567, *p* = 0.033). **(B)** The CSS curve of stage I NSCLC patients who received thermal ablation or wedge resection after PSM (χ^2^ = 4.297, *p* = 0.038).

After 1:1 matching, the KM curves for patients with different treatment were obtained (Figure 2). The OS of patients with wedge resection were still better than those with thermal ablation after PSM (log rank test, *p* = 0.033) (Figure 2A). The estimated 3- and 5-year OS rates were 64.3 and 44.5%, respectively, in the wedge resection group, with a median OS of 53 months (95%CI: 39–50); the corresponding rates were 51.6 and 30.1% in the thermal ablation group, with a median OS of 37 months (95%CI: 28–46). The CSS of patients in the wedge resection group was also better than those in the thermal ablation group (log rank test, *p* = 0.038) (Figure 2B). The estimated 3- and 5-year CSS rates were 75.2 and 63.5%, respectively, in the wedge resection group, with a median CSS of 132 months (95%CI: 59–204), the corresponding rates were 62.6 and 46.0% in the thermal ablation group, with a median OS of 50 months (95%CI: 23–76). The results of the Cox analysis of OS and CSS after PSM are shown in Table 3. Thermal ablation was no longer a risk factor for OS or CSS when compared with wedge resection. Patients who received radiation had worse OS (HR: 1.849, 95%CI: 1.132–3.019) and CSS (HR: 1.879, 95%CI: 1.030–3.425). Co-variables such as race and tumor size were associated with OS. Variable tumor size was associated with CSS.

**TABLE 3:** Univariate and multivariate Cox regression of OS and CSS in stage I NSCLC patients having thermal ablation and wedge resection after PSM.

	Overall survival				Cancer specific survival			
	Univariate		Multivariate		Univariate		Multivariate	
Variables	HR (95%CI)	*P*-value	HR (95%CI)	*P*-value	HR (95%CI)	*P*-value	HR (95%CI)	*P*-value
**Age**								
≤75	Reference				Reference			
>75	1.031 (0.755−1.409)	0.846			0.963 (0.638−1.453)	0.858		
**Sex**								
Female	Reference				Reference			
Male	1.191 (0.870−1.630)	0.275			0.942 (0.615−1.441)	0.782		
**Race**								
Others	Reference				Reference			
White	0.330 (0.081−1.346)	0.122	0.289 (0.067−1.255)	0.098	0.229 (0.056−0.943)	0.041	0.278 (0.061−1.272)	0.099
Black	0.382 (0.085−1.712)	0.209	0.311 (0.065−1.487)	0.143	0.221 (0.044−1.104)	0.066	0.260 (0.046−1.474)	0.128
Asian or Pacific islander	0.105 (0.017−0.637)	0.014	0.086 (0.014−0.541)	0.009	0.020 (0.095−0.686)	0.020	0.094 (0.012−0.721)	0.023
**Marital status**								
No	Reference				Reference			
Yes	1.083 (0.788−1.487)	0.624			1.045 (0.686−1.592)	0.838		
**Side**								
Left	Reference				Reference			
Right	0.923 (0.675−1.263)	0.617			0.687 (0.455−1.037)	0.074		
**Site**								
Unknown	Reference				Reference			
Upper lobe	1.383 (0.507−3.777)	0.527			1.535 (0.373−6.318)	0.553		
Lower lobe	1.295 (0.469−3.547)	0.618			1.369 (0.327−5.725)	0.667		
Middle lobe	1.537 (0.472−4.999)	0.475			1.551 (0.300−8.008)	0.601		
**Tumor size**								
>2 cm	Reference				Reference			
≤1 cm	0.649 (0.356−1.182)	0.157	0.614 (0.331−1.141)	0.123	0.663 (0.298−1.477)	0.314	0.614 (0.266−1.417)	0.253
>1 cm and ≤2 cm	0.728 (0.527−1.007)	0.055	0.681 (0.487−0.954)	0.025	0.675 (0.440−1.037)	0.073	0.621 (0.393−0.979)	0.040
**Histology**								
Others	Reference				Reference			
Squamous cell carcinoma	1.012 (0.640−1.601)	0.959			1.066 (0.582−1.952)	0.836		
Adenocarcinoma	0.796 (0.512−1.238)	0.310			0.753 (0.416−1.365)	0.350		
**Grade**								
Unknown	Reference				Reference			
Grade I/II	0.774 (0.534−1.123)	0.177			0.563 (0.335−0.946)	0.030	0.666 (0.389−1.141)	0.139
Grade III/IV	0.924 (0.627−1.361)	0.688			0.819 (0.491−1.365)	0.443	0.842 (0.488−1.455)	0.539
**Lymph node resection**								
No	Reference				Reference			
Yes	0.687 (0.337−1.399)	0.301			0.290 (0.071−1.180)	0.084	0.284 (0.068−1.187)	0.085
**Adjuvant therapy**								
No	Reference				Reference			
Radiation	1.849 (1.132−3.019)	0.014	1.685 (1.026−2.767)	0.039	2.322 (1.293−4.171)	0.005	1.879 (1.030−3.425)	0.040
Chemotherapy	0.821 (0.416−1.620)	0.569	0.718 (0.348−1.482)	0.370	1.272 (0.580−2.787)	0.549	0.943 (0.401−2.219	0.893
Both	1.490 (0.780−2.847)	0.228	1.241 (0.640−2.408)	0.522	2.158 (1.031−4.516)	0.041	1.529 (0.698−3.353)	0.289
**Treatment**								
Wedge resection	Reference				Reference			
Thermal ablation	1.401 (1.025−1.916)	0.035	1.359 (0.983−1.879)	0.064	1.546 (1.019−2.345)	0.041	1.645 (1.062−2.549)	0.076

*PSM, propensity score matching; OS, overall survival; CSS, cancer specific survival; HR, hazard ratio; CI, confidence interval.*

### Subgroup Analysis

**FIGURE 3:**
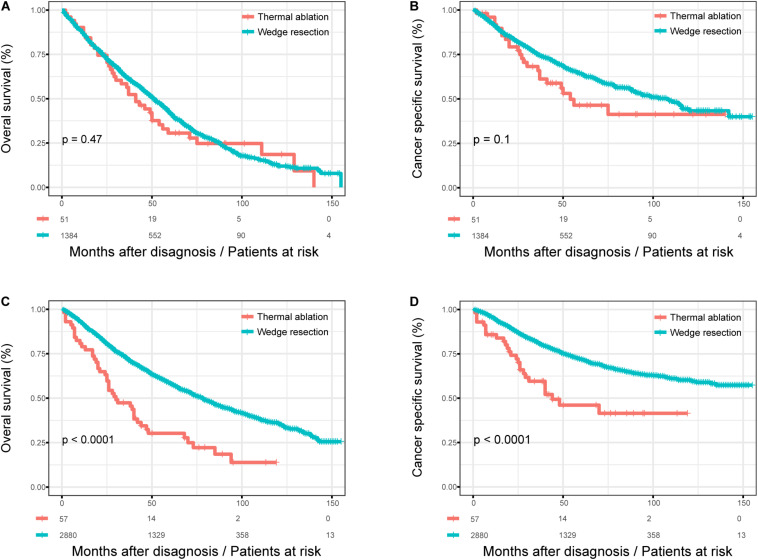
The subgroup analysis of OS and CSS in stage I NSCLC patients who received thermal ablation or wedge resection. **(A)** The OS curve of stage I NSCLC patients aged >75 years who received thermal ablation or wedge resection (χ^2^ = 0.515, *p* = 0.47). **(B)** The CSS curve of stage I NSCLC patients who received thermal ablation or wedge resection aged over 75 years (χ^2^ = 2.708, *p* = 0.1). **(C)** The OS curve of stage I NSCLC patients <75 years who received thermal ablation or wedge resection (χ^2^ = 32.190, *p* < 0.0001). **(D)** The CSS curve of stage I NSCLC patients <75 years who received thermal ablation or wedge resection (χ^2^ = 20.966, *p* < 0.0001).

We studied the influence of the surgical method on OS and CSS in patients according to their age at diagnosis before and after PSM. Figure 3 shows the Kaplan–Meier survival curves for patients with different age at diagnosis before PSM. There was no significant difference in OS (log rank test, *p* = 0.470) and CSS (log rank test, *p* = 0.100) between the thermal ablation and wedge resection groups when age at diagnosis was >75 years (Figures 3A,B). The 5-year OS and CSS rates were 30.6 and 46.4%, respectively, for those with thermal ablation; the corresponding values were 41.7 and 64.1% for those with wedge resection. However, patients with wedge resection had better OS (log rank test, *p* < 0.0001) and CSS (log rank test, *p* < 0.0001) when age at diagnosis was <75 years (Figures 3C,D). The 5-year OS and CSS rates were 30.2 and 46.1%, respectively, for those with thermal ablation; the corresponding rates were 58.3 and 71.9% for those with wedge resection.

### Subgroup Analysis After PSM

**FIGURE 4:**
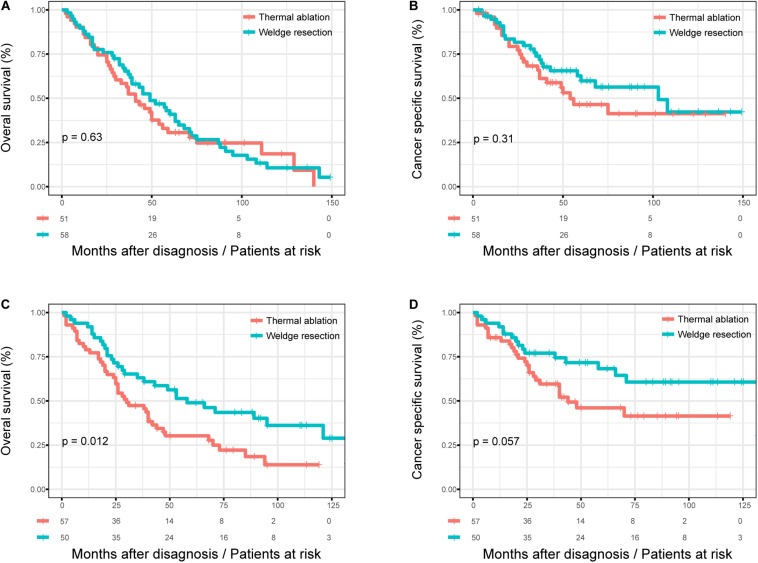
The subgroup analysis of OS and CSS in stage I NSCLC patients who received thermal ablation or wedge resection. **(A)** The OS curve of stage I NSCLC patients >75 years who received thermal ablation or wedge resection after PSM (χ^2^ = 0.233, *p* = 0.63). **(B)** The CSS curve of stage I NSCLC patients >75 years who received thermal ablation or wedge resection after PSM (χ^2^ = 1.04, *p* = 0.31). **(C)** The OS curve of stage I NSCLC patients <75 years who received thermal ablation or wedge resection after PSM (χ^2^ = 6.384, *p* = 0.012). **(D)** The CSS curve of stage I NSCLC patients <75 years who received thermal ablation or wedge resection after PSM (χ^2^ = 3.633, *p* = 0.057).

Figure 4 shows the KM survival curves for patients with different age at diagnosis after PSM. There was still no significant difference in OS (log rank test, *p* = 0.470) and CSS (log rank test, *p* = 0.100) between the thermal ablation and wedge resection groups after PSM when age at diagnosis was >75 years (Figures 4A,B). The 5-year OS and CSS rates were 30.6 and 46.4%, respectively, for those with thermal ablation; the corresponding values were 41.0 and 59.8% for those with wedge resection. Patients in the wedge resection group had better OS than those in the thermal ablation group (log rank test, *p* < 0.0001), but no significant difference was noted between the two groups with respect to CSS (log rank test, *p* = 0.057), when age at diagnosis was <75 years (Figures 4C,D). The 5-year OS and CSS rates were 30.2 and 46.1%, respectively, for those with thermal ablation; the corresponding rates were 48.9 and 68.2% for those with wedge resection.

## Discussion

In recent years, the detection rate of pulmonary nodules has increased owing to high-resolution computed tomography (CT) enabling the detection of early-stage lung cancer (14). Although surgery is the standard of treatment for early stage patients, there were other therapies to choose from. Lobectomy is a standard surgical procedure for stage I NSCLC (1). For those who cannot tolerate lobectomy, segmentectomy and wedge resection can be considered. Although wedge resection is a non-anatomical resection, several studies have shown wedge resection to be beneficial in stage I NSCLC when the tumor size is small (15, 16). However, patients who are older or have multiple morbidities may have reduced cardiopulmonary function after surgical resection. Local-treatment options such as stereotactic body radiation therapy (SBRT) and thermal ablation have been regarded as viable alternatives for patients that are unfit for surgery (17–19). Multiple trials have shown that SBRT in treating early-stage NSCLC in elderly people is safe and effective. For elderly people with a greater number of comorbidities, SBRT has proven to be a curative modality, with comparable local tumor control and 3-year OS rates to lobectomy (20–23). On the other hand, Chaitan et al. (24) suggested that thermal ablation could be used to effectively treat or control stage IA NSCLC in inoperable patients (3-year OS, 50%). However, few studies have investigated whether thermal ablation is adequate for stage I NSCLC compared with wedge resection when lobectomy or segmentectomy is unfit for patients or for those unwilling to undergo invasive surgery. In this study, we compared the outcome of patients undergoing wedge resection or thermal ablation. We also explored whether thermal ablation is an option for those who cannot undergo lobectomy but can tolerate wedge resection.

In this study, before PSM, we found that the thermal ablation group had worse OS and CSS than the wedge resection group. Apart from the surgery method, seven factors including age, sex, tumor size, histology, grade, lymph node resection, and adjuvant therapy were found to be associated with OS and CSS outcomes in the multivariate Cox regression analysis. We performed subgroup survival analysis, and found that thermal ablation had comparable OS and CSS to wedge resection, when patients were >75 years old. After PSM, the thermal ablation group was no longer a risk factor of OS or CSS. Patients who received radiation had worse OS and CSS than those who did not. The other two factors including ethnicity and tumor size were found to be associated with OS and CSS outcomes in the multivariate Cox regression. However, upon subgroup analysis after PSM, thermal ablation was still equivalent to wedge resection with respect to OS and CSS in patients >75 years old. The number of comorbidities increases with age (25), and elderly NSCLC patients would likely benefit from thermal ablation rather than surgery. Several studies have also revealed that thermal ablation showed safety and efficacy in both primary and secondary lung malignancies, and after propensity score matching, the 2-year OS survival rates could be comparable with surgery and SBRT (26, 27). Moreover, our large database study suggested that compared with wedge resection, thermal ablation may be the optimal procedure for patients >75 years with stage I NSCLC, based on the 8th edition of TNM classification. However, for patients <75 years who are unfit for lobectomy, we still recommend wedge resection, provided they can tolerate it.

To our best knowledge, this is the first database study assessing the equivalency of thermal ablation vs. wedge resection in stage I NSCLC based on the 8th edition of TNM classification. This study used a multicenter population-based database and included 4,372 patients with precise long-term survival data. We used several statistical methods including PSM, Cox regression analysis, and KM curves to confirm the results. This study also revealed the current treatment for stage I NSCLC patients who are unfit for radical surgery in the real word.

Our study has some limitations. Firstly, thermal ablation is offered to patients with stage I NSCLC who are unfit for surgical resection because of cardiorespiratory comorbidity or insufficient vital lung function (28). However, information on multiple comorbidities and cardio-pulmonary function is not available in the SEER database. The reason for these patients choosing thermal ablation is not known, which may cause a degree of bias. Secondly, information regarding tumor grade was unknown in some of the patients, which may further bias our results. Thirdly, this study is a retrospective study with inevitable intrinsic shortcomings. Despite these limitations, we believe that our findings may assist future research designs and proposals.

## Conclusion

In conclusion, this study demonstrated that for stage I NSCLC, thermal ablation could be a potential option for patients unfit for lobectomy, especially those aged >75 years. We still recommended wedge resection for patients who are <75 years. More studies are needed to investigate the equivalency of thermal ablation vs. wedge resection in stage I NSCLC.

## Data Availability Statement

Publicly available datasets were analyzed in this study. This data can be found here: Surveillance, Epidemiology, and End Results (SEER) database (https://seer.cancer.gov/).

## Author Contributions

XF and YT designed and directed the project. CZ and JL planned and carried out the majority of the project and took the lead in writing the manuscript. All authors contributed to the article and approved the submitted version.

## Conflict of Interest

The authors declare that the research was conducted in the absence of any commercial or financial relationships that could be construed as a potential conflict of interest.
